# Continuous renal replacement therapy using a cellulose triacetate hemofilter for severe coronavirus disease

**DOI:** 10.1186/s41100-022-00436-1

**Published:** 2022-09-05

**Authors:** Kanako Takahashi, Hiroyuki Inoue, Masumi Kishimoto, Ryuichi Nakayama, Takehiko Kasai, Naofumi Bunya, Keisuke Harada, Shuji Uemura, Eichi Narimatsu

**Affiliations:** 1 Department of Emergency Medicine, Sapporo Medical University, 16-291, Minami-1-jo Nishi, Chuo-ku, Sapporo-shi, Hokkaido 060-8543 Japan; 2 Division of Clinical Engineering, Sapporo Medical University Hospital, Sapporo-shi, Japan

**Keywords:** Renal replacement therapy, COVID-19, ARDS, Cytokine, Cellulose triacetate filter, Interleukin-6

## Abstract

**Background:**

In patients with severe coronavirus disease (COVID-19), the use of acrylonitrile hemofilters can reduce cytokine concentrations. However, acrylonitrile hemofilters can easily coagulate, and the effect of hemofilters on improvement in patient prognosis remains unclear. Therefore, we aimed to investigate the changes in serum cytokine concentrations, alleviation of organ damage, and improvement in patient prognosis with continuous renal replacement therapy (CRRT) using a cellulose triacetate (CTA) filter with excellent anticoagulation property in patients with severe COVID-19.

**Methods:**

This was a retrospective, single-center study conducted by the Advanced Critical Care Center in Sapporo Medical University Hospital, Japan. Seven patients with severe COVID-19 between March 01 and June 30, 2020, were included. The patients were under mechanical ventilation and received continuous blood purification therapy with a CTA filter. We summarized the CRRT status and patient prognosis and measured their serum cytokine (interleukin [IL]-1*β*, IL-4, IL-6, IL-8, IL-10, tumor necrosis factor-*α*, and interferon-*γ*) and serum marker levels, before and after CRRT. In addition, we evaluated the changes in their respiratory status, hemodynamics, and organ dysfunction scores. The average age of the patients was 61.5 years, and five patients were male. Extracorporeal membrane oxygenation was used in five patients. The treatment outcome included three deaths.

**Results:**

The median CRRT duration was 7 days. The hemofilter was replaced once a day. After CRRT, the IL-6 concentration decreased from 393 to 85 pg/mL (*p* = 0.016), the Krebs von den Lungen-6 concentration decreased from 554 to 350 U/mL, and the PaO_2_/FiO_2_ ratio increased significantly from 90 to 248, and therefore, oxygenation improved. In addition, the norepinephrine dose and lactate level decreased, and the circulation tended to improve; however, the renal function and Sequential Organ Failure Assessment score did not change.

**Conclusions:**

The serum IL-6 level decreased, and the respiratory status improved upon CRRT using a CTA filter in patients with severe COVID-19.

## Background

Coronavirus disease (COVID-19), an infectious disease caused by severe acute respiratory syndrome coronavirus 2, is known to be severe in approximately 10% of the patients and is associated with a high mortality rate of 30–40% [1]. In patients with severe COVID-19, a cytokine storm caused by excessive production of inflammatory cytokines has been reported to cause organ damage via endothelial cell injury [2].

The lung, kidney, and blood cells, which are the main targets of hypercytokinemia, are considered to be involved in COVID-19 pathology. In patients with severe COVID-19 experiencing acute respiratory distress syndrome (ARDS), the serum level of interleukin (IL)-6, one of the inflammatory cytokines, is negatively correlated with the PaO2/FiO2 ratio. An elevated IL-6 level is reportedly associated with ARDS severity [3]. In addition, an increase in Krebs von den Lungen (KL)-6 level is not only a prognostic factor for ARDS, but also a predictor of COVID-19 severity [4]. In severe COVID-19, acute kidney injury (AKI) has also been reported in 20–40% of cases [5], indicating a poor prognosis [6]. Furthermore, extracorporeal membrane oxygenation (ECMO) introduced for treating severe respiratory failure due to COVID-19 is considered a potential trigger of cytokine storms [7].

In general, blood purification therapy for AKI, associated with bacterial sepsis or multiple organ dysfunction, has been conventionally used to remove uremic substances and inflammatory mediators. Several patients have been treated using poly methyl methacrylate and acrylonitrile filters [8–10]. Moreover, in patients with severe COVID-19, the use of acrylonitrile hemofilters has been reported to reduce cytokine concentrations [11]. However, the effect of hemofilters in improving patient prognosis remains unclear, and acrylonitrile hemofilters have the disadvantage of being easily coagulated [12].

As COVID-19 is a contagious infectious disease, it is recommended to replace the hemofilter less frequently to reduce the risk of infection to medical staff. However, to the best of our knowledge, there are no reports on the application of a cellulose triacetate (CTA) hemofilter, which has excellent anticoagulant properties and a long life [13]. Therefore, in this study, we aimed to investigate the changes in serum cytokine concentrations and other parameters upon continuous renal replacement therapy (CRRT), a type of blood purification therapy, using a CTA filter in patients with severe COVID-19 and determine its effect on prognosis.

## Methods

### Study design and patients

This retrospective study involved 11 patients who were admitted to the Advanced Critical Care Center in Sapporo Medical University for 3 months (from March 01 to June 30, 2020) and diagnosed with COVID-19 using an RT-PCR test for severe acute respiratory syndrome coronavirus 2. All patients were under ventilation and were administered CRRT using a CTA filter. Finally, seven patients were included in this study based on the published opt-out policy, and the remaining four patients with insufficient data and samples were excluded. The study was approved by the institutional review board of Sapporo Medical University. All study procedures were performed in accordance with the tenets of the Declaration of Helsinki.

## Materials

We used ACH-Σ^®^ (Asahi Kasei Medical, Tokyo, Japan) for blood purification therapy and CHDF-SG^®^ (Asahi Kasei Medical) for maintaining the blood circuit. The selected treatment mode was continuous veno-venous hemofiltration, and the hemofilter used was UT-1100S^®^ or UT-2100S^®^ (NIPRO, Tokyo, Japan). The setting of the membrane area and conditions was left to the discretion of the attending physician. The blood flow rate was set to 150 mL/min, blood purification amount to the replacement flow rate was 1000–2500 mL/min, and the filtrate amount was set appropriately according to the amount of water removed. Subpack^®^ (NIPRO) was used as the replacement solution. Heparin or nafamostat mesylate was used as the anticoagulant, and the activated partial thromboplastin time was controlled in 40–60 s. However, in patients who underwent veno-venous-ECMO, anticoagulants were not used when the activated partial thromboplastin time was prolonged. The femoral vein or internal jugular vein was selected for vascular access using the emergency blood access catheter 13 Fr Power-Trialysis^®^ (Medicon, Osaka, Japan).

### Clinical data collection

Using the electronic medical records of the participants, we extracted data regarding patient background, treatment course, and prognosis. Arterial blood samples collected from the patients were dispensed into biochemical blood-collection tubes, and after leaving undisturbed for some time, the sample tubes were centrifuged at 3000 rpm for 5 min, and the serum was dispensed into a micro spitz tube for further analyses. Serum IL-1*β*, IL-4, IL-6, IL-8, IL-10, interferon (IFN)-*γ*, and tumor necrosis factor (TNF)-*α* levels were measured at the time of introduction (before) and on day 7 after the blood purification therapy, using the respective enzyme-linked immunosorbent assay kits (SRL Inc., Tokyo, Japan), according to the manufacturer’s instructions.

Additionally, C-reactive protein, procalcitonin, ferritin, sialylated carbohydrate antigen KL-6, creatinine, and D-dimer levels were determined before and after blood purification therapy. We also conducted a blood gas analysis and measured PaO_2_/FiO_2_, lung compensation, heart rate, mean arterial pressure, and norepinephrine level. In addition, the Sequential Organ Failure Assessment score was calculated.

### Statistical analysis

Parameters such as median value, interquartile range, and the ratio of each item were calculated. For the paired value comparison (before and after therapy), Wilcoxon signed rank-sum test was performed, and results with *p* < 0.05 were considered significant.

All statistical analyses were performed using EZR version 1.53, a statistical software that extends the functionality of the R and R Commander and is freely available online at Jichi Medical University Hospital, Saitama Medical Center (Tochigi, Japan) [14].

## Results

Table 1 shows the clinical characteristics of the patients with COVID-19 who were on CRRT using a CTA filter. The average age of the seven patients was 61.6 ± 9.5 years, and five patients were men. Mechanical ventilation was required for all patients; five of the seven patients also underwent ECMO. The AKI stage [15] at the beginning of CRRT was 1 in two patients, 2 in three patients, and 3 in two patients. Continuous veno-venous hemofiltration was selected for all cases, with a median treatment duration of 7 days. As an anticoagulant, nafamostat mesylate was administered to six patients and heparin to one patient. Three patients succumbed to the disease after leaving the intensive care unit of the hospital.

**Table 1:** Clinical characteristics of patients treated with continuous renal replacement using a cellulose triacetate filter

Age	years, mean (SD)	61.6 (9.5)
Male	*n* (%)	5 (71.4)
Acute kidney injury stage	*n* (%)	
	Stage 1	2 (28.6)
	Stage 2	3 (42.9)
	Stage 3	2 (28.6)
Hemofilter	*n* (%)	
	UT-2100S	6 (85.7)
	UT-1100S	1 (14.3)
Anticoagulant	*n* (%)	
	Nafamostat mesylate	6 (85.7)
	Heparin	1 (14.3)
Therapy onset post admission	h, median (IQR)	7 (5–89)
Dosage	mL/kg/h, median (IQR)	1000 (1000–2000)
Duration	days, median (IQR)	7 (6–9)
Circuit change	times/day, median (IQR)	1.0 (0.5–1.5)
with ECMO	*n* (%)	5 (71.4)
Outcome	*n* (%)	
	Survived	4 (57.1)

*ECMO* extracorporeal membrane oxygenation, *IQR* interquartile range

**Fig. 1:**
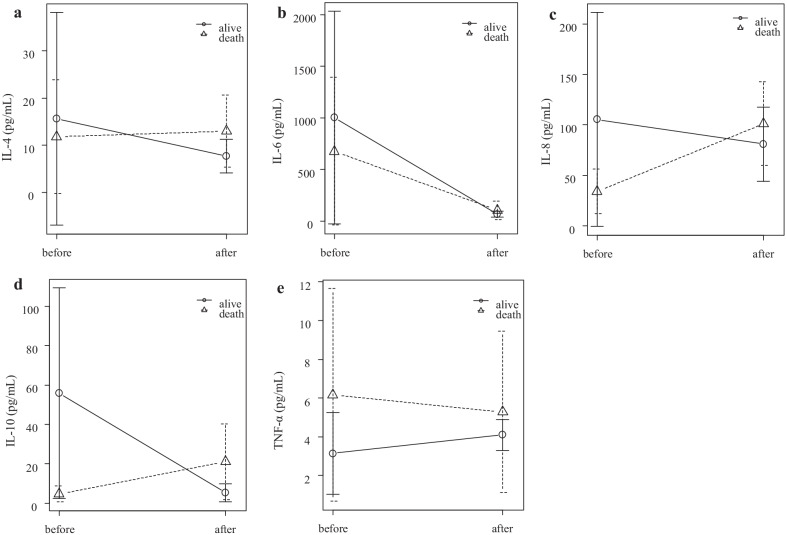
Serum cytokine concentrations before and after continuous renal replacement therapy. The serum IL-6 concentration (**b**) significantly reduced after therapy (*p* = 0.016). The serum IL-4 (**a**), IL-8 (**c**), IL-10 (**d**), and TNF-*α* (**e**) concentrations showed no significant changes after therapy. Mean ± SD.IL, interleukin; TNF, tumor necrosis factor; CRRT, continuous renal replacement therapy

The serum cytokine concentrations before and after CRRT are shown in Table 2. The IL-6 concentration significantly reduced after CRRT. The changes in the serum cytokine concentrations after CRRT in each patient are shown in Fig. 1. After CRRT, the KL-6 concentration and PaO2/FiO2 ratio significantly increased compared with those before CRRT. Furthermore, the mean arterial pressure and norepinephrine dose decreased after the treatment (Table 2).

**Table 2:** Other clinical parameters before and after continuous renal replacement therapy

Parameter	Before therapy	After therapy	*p*
	Median [IQR]	Median [IQR]	
C-reactive protein (mg/dL)	15.3 [5.4–30.3]	8.2 [5.9–13.5]	0.219
Procalcitonin (ng/mL)	0.96 [0.36–30.34]	0.89 [0.46–1.49]	0.156
Ferritin (ng/mL)	605 [549–1302]	924 [721–1502]	0.813
KL-6 (U/mL)	554 [329–800]	350 [249–553]	0.016
PaO_2_ /FiO_2_	90 [79–153]	248 [195–274]	0.016
Compliance (mL/cmH_2_O)	32 [20–40]	31 [27–59]	0.375
Creatinine (mg/dL)	1.14 [0.86–1.35]	1.00 [0.94–1.43]	0.688
D-dimer (μg/mL)	11.7 [4.6–21.1]	8.3 [6.9–8.8]	0.813
Heart rate (beat/min)	101 [83–107]	113 [100–119]	0.156
Mean arterial pressure (mmHg)	82 [69–96]	57 [47–66]	0.016
Norepinephrine (μg/kg/min)	0.10 [0.04–0.16]	0	0.058
Lactate (mmol/L)	1.4 [1.1–2.2]	1.2 [1.1–1.4]	0.078
Sequential organ failure assessment score	13 [10–14]	11 [9–12]	0.169

*IQR* interquartile range

## Discussion

In this study, we evaluated the changes in inflammatory cytokine concentrations in patients with COVID-19 who had received CRRT using a CTA filter. We found that the serum IL-6 concentration significantly decreased after CRRT.

Cytokine storms have been reported to be involved in the pathology of COVID-19, especially the severe form [16]. A cytokine storm is a systemic inflammatory response involving the release of a series of cytokines such as TNF-*α*, IL-1*β*, IL-2, IL-6, IFN-*α*, and monocyte chemoattractant protein-1. These cytokines stimulate immune cells to release large amounts of free radicals and are a major cause of ARDS and multiple organ failure syndrome. In particular, attention has been paid to the correlation between COVID-19 severity and IL-6 level. It has been reported that the COVID-19-related mortality rate reduces with a decrease in the serum IL-6 concentration [17].

According to Ronco et al., CRRT does not eliminate the basal levels of cytokines; instead, it is regarded as an adjunct therapy to remove excess cytokines, as defined by the peak concentration hypothesis, to provide benefits to the patients [18]. Although the levels of inflammatory cytokines in patients with COVID-19 are lower than those in other diseases such as sepsis, trauma, and out-of-hospital cardiac arrest [19], they were higher in this study than in previous studies, especially the level of IL-6. Nonetheless, there is no contradiction because the disease condition ameliorated upon suppression of the peak cytokine concentration using CRRT with a CTA filter.

According to Honore et al., in the pathophysiology of hypercytokine storms, there is an imbalance in the levels of mediators, with certain cytokines accounting for a large proportion of the cytokine storm [20]. In this study, the levels of cytokines other than IL-6 tended to decrease after CRRT, but no significant difference was observed. This could be attributed to the fact that the IL-6 level in the blood was relatively high, and the rate of removal of other cytokines was low.

Blood purification therapy requires bedside work, including circuit attachment/detachment and catheter management. During COVID-19 treatment, medical staff should work in a controlled infected area; the lowest number of individuals working in the shortest time interval has been sought to reduce the risk of infection [21]. Thus, the advantages of selecting a CTA filter are its excellent anticoagulation properties and a long life compared with those of the acrylonitrile filters that require frequent replacement and coagulate easily. UT Filter^®^ is a continuous slow hemofiltration device that uses a CTA filter of a uniform cross section. The filter has a long life and is antithrombotic, with the ability to remove inflammatory cytokines via filtration [22]. Polysulfone filters are usually the first choice at our hospital. However, abnormal coagulation with these filters has been reported, characteristically in the cases of COVID-19 [17]. Therefore, we selected a CTA filter with excellent antithrombotic properties; coagulation was less likely when using a CTA filter. In addition, it was possible to remove cytokines such as IL-6 through long-term CRRT [23]. In this study, veno-venous-ECMO was performed simultaneously in five out of the seven patients. Two of the seven patients manifested hemorrhagic events, such as epistaxis, which were not related to the blood purification therapy since its initiation. Therefore, only a small amount of an anticoagulant was administered to these patients, and in-circuit coagulation was frequently observed, with the circuit being replaced up to four times a day. However, there were no catheter problems in these patients. In the other five patients, the anticoagulant dose was lower than usual, but CRRT could be performed with regular 24-h circuit changes. Thus, it was considered that the selection of the CTA filter was effective. Nafamostat mesylate is a serine protease inhibitor that strongly inhibits proteolytic enzymes, such as thrombin, plasmin, and trypsin. In Japanese patients, it is often effective in alleviating fibrinolytic DIC. Patients with severe COVID-19 exhibit the characteristics of fibrinolytic DIC; therefore, nafamostat is considered promising. We used nafamostat mesylate in most cases for alleviating coagulation within the CRRT circuit because of the risk of unexpected bleeding. This ensured effective CRRT [24].

In COVID-19, there is a negative correlation between the IL-6 level and PaO2/FiO2 ratio, which is reportedly different from that in other types of ARDS [3]. The reason for this is that the invasive cytokine IL-6 has a key role in the initial inflammatory process. In this study, after CRRT, the IL-6 and KL-6 (a prognostic factor for ARDS) levels significantly decreased, whereas the PaO2/FiO2 ratio significantly increased. A decrease in the IL-6 level is likely to have contributed to the amelioration of ARDS.

Blood purification therapy for hypercytokinemia has been reported to be effective in improving hemodynamics in diseases such as sepsis [25]. Removal of excess cytokines from the blood may repair the barrier function of the vascular endothelium and improve circulation [26]. In this study, the dose of norepinephrine was reduced after CRRT.

Nonetheless, our study had a few limitations. First, we did not conduct a comparative analysis using other filters or a patient group that did not receive CRRT. Second, our study was a single-center retrospective study with a small number of patients. Finally, the timing and conditions for CRRT were at the discretion of the physician in charge. In the future, studies with a higher number of critically ill patients with COVID-19 who require blood purification therapy are needed.

## Conclusions

Here, patients with severe COVID-19 received CRRT using a CTA filter; the serum IL-6 level significantly decreased, and the respiratory status improved after CRRT. Although the cytokine-removal effect of CRRT was expected, CTA filters with a long membrane life could be candidates to achieve infection control in the clinical setting.

## Data Availability

All data generated or analyzed during this study are included in this published article.

## Abbreviations

COVID-19: Coronavirus disease 2019
CRRT: Continuous renal replacement therapy
CTA: Cellulose triacetate
IL: Interleukin
ARDS: Acute respiratory distress syndrome
KL-6: Krebs von den Lungen-6
AKI: Acute kidney injury
ECMO: Extracorporeal membrane oxygenation
IFN: Interferon
TNF: Tumor necrosis factor
